# “*Candidatus* Subterrananammoxibiaceae,” a New Anammox Bacterial Family in Globally Distributed Marine and Terrestrial Subsurfaces

**DOI:** 10.1128/aem.00800-23

**Published:** 2023-07-20

**Authors:** Rui Zhao, Sven Le Moine Bauer, Andrew R. Babbin

**Affiliations:** a Department of Earth, Atmospheric and Planetary Sciences, Massachusetts Institute of Technology, Cambridge, Massachusetts, USA; b Centre for Deep Sea Research, Department of Earth Science, University of Bergen, Bergen, Norway; Colorado School of Mines

**Keywords:** anammox bacteria, marine sediments, metagenome, novel lineage, terrestrial subsurface

## Abstract

Bacteria specialized in anaerobic ammonium oxidation (anammox) are widespread in many anoxic habitats and form an important functional guild in the global nitrogen cycle by consuming bio-available nitrogen for energy rather than biomass production. Due to their slow growth rates, cultivation-independent approaches have been used to decipher their diversity across environments. However, their full diversity has not been well recognized. Here, we report a new family of putative anammox bacteria, “*Candidatus* Subterrananammoxibiaceae,” existing in the globally distributed terrestrial and marine subsurface (groundwater and sediments of estuary, deep-sea, and hadal trenches). We recovered a high-quality metagenome-assembled genome of this family, tentatively named “*Candidatus* Subterrananammoxibius californiae,” from a California groundwater site. The “*Ca.* Subterrananammoxibius californiae” genome not only contains genes for all essential components of anammox metabolism (e.g., hydrazine synthase, hydrazine oxidoreductase, nitrite reductase, and nitrite oxidoreductase) but also has the capacity for urea hydrolysis. In an Arctic ridge sediment core where redox zonation is well resolved, “*Ca.* Subterrananammoxibiaceae” is confined within the nitrate-ammonium transition zone where the anammox rate maximum occurs, providing environmental proof of the anammox activity of this new family. Phylogenetic analysis of nitrite oxidoreductase suggests that a horizontal transfer facilitated the spreading of the nitrite oxidation capacity between anammox bacteria (in the *Planctomycetota* phylum) and nitrite-oxidizing bacteria from *Nitrospirota* and *Nitrospinota*. By recognizing this new anammox family, we propose that all lineages within the “*Ca.* Brocadiales” order have anammox capacity.

**IMPORTANCE** Microorganisms called anammox bacteria are efficient in removing bioavailable nitrogen from many natural and human-made environments. They exist in almost every anoxic habitat where both ammonium and nitrate/nitrite are present. However, only a few anammox bacteria have been cultured in laboratory settings, and their full phylogenetic diversity has not been recognized. Here, we present a new bacterial family whose members are present across both the terrestrial and marine subsurface. By reconstructing a high-quality genome from the groundwater environment, we demonstrate that this family has all critical enzymes of anammox metabolism and, notably, also urea utilization. This bacterium family in marine sediments is also preferably present in the niche where the anammox process occurs. These findings suggest that this novel family, named “*Candidatus* Subterrananammoxibiaceae,” is an overlooked group of anammox bacteria, which should have impacts on nitrogen cycling in a range of environments.

## INTRODUCTION

Bacteria specialized in anaerobic ammonium oxidation (anammox) play a critical role in the nitrogen cycle in marine environments. By converting nitrite and ammonium to dinitrogen gas, they are thought to be responsible for 30% to 70% of the total N_2_ released into the atmosphere (1, 2) and can be particularly important in some natural environments (e.g., 3–5). They can also significantly modulate the distribution of nitrite in marine sediments (6). Resolving the full diversity of anammox bacteria is critical to deciphering their environmental impacts and evolutionary significance, e.g., their emergence time on early Earth (7).

Anammox bacteria were first discovered in a denitrifying fluidized-bed reactor in 1995 (8–10). Although they are widespread in many anoxic habitats, no anammox bacterium has yet been isolated. Rather, well-established cultures of anammox bacteria are enrichments obtained mainly from engineering environments (11–15) and from coastal sediments (16, 17). Biochemical, genomic, and transcriptomic studies of these enrichment cultures have revealed that the most unique enzyme (and one exclusive to anammox based on current knowledge) in these bacteria is the hydrazine synthase, which combines ammonium and nitric oxide to produce hydrazine (18, 19) in a specialized cell compartment called the anammoxosome. The produced hydrazine does not accumulate in anammox bacteria but, rather, is oxidized to N_2_ by hydrazine dehydrogenase (20). The two essential substrates of anammox bacteria, ammonium and nitrite, are thought to be derived from the extracellular environment, although some marine anammox bacteria are capable of generating ammonium from urea hydrolysis intracellularly (4, 21). Anammox bacteria reduce nitrite to nitric oxide to fuel the hydrazine synthesis and can also oxidize it to nitrate to fix carbon (19). These coupled reactions form the core metabolism of anammox bacteria. Phylogenetically, the enrichment cultures of anammox bacteria are predominantly affiliated with two families: “*Candidatus* Brocadiaceae” and “*Candidatus* Scalinduaceae” (22).

The diversity of anammox bacteria in the environment is greater than those represented by the enrichment cultures. For example, some genomes of uncultured anammox bacteria of the two known families (“*Ca.* Scalinduaceae” and “*Ca.* Brocadiaceae”) have been recovered from deep brines (23, 24) and wastewater treatment plants (12). Recently, through genome reconstruction, two new families of potential anammox bacteria were discovered (i.e., “*Candidatus* Anammoxibacteraceae,” found in subsea tunnel biofilms [25], and “*Candidatus* Bathyanammoxibiaceae,” found in marine sediments and groundwater [26]). Bacterial genomes of these two families contain genes encoding the above-described key and diagnostic anammox enzymes and have similar distribution patterns as the known anammox bacteria (26), suggesting that they are all anammox bacteria. Therefore, a total of four putative anammox bacterial families have been identified so far, all of which are within the *Brocadiales* order in the *Planctomycetota* phylum. However, whether these four families represent the entire diversity of anammox bacteria is still unknown.

In the current version of the Genome Taxonomy Database (GTDB, 08-RS124), there is an understudied family (with the placeholder JACQHT01) within the *Brocadiales* order that contains only two metagenome-assembled genomes (MAGs) recovered from the California groundwater environment (27). Because these two MAGs are only partial (<71% complete) and lack the diagnostic hydrazine synthase genes, they are not counted as anammox bacteria. Therefore, it remains unknown whether anammox bacteria can be affiliated with lineages beyond the currently known four families and whether all *Brocadiales* maintain the anammox function.

In this study, by 16S rRNA gene phylogenetic analysis, we first reveal an unstudied family matching f__JACQHT01 within the *Brocadiales* order, which exists in both the marine and terrestrial subsurface. To examine the potential functions of this new family, we leveraged the metagenome sequencing data from a California groundwater site to recover the first high-quality genome of this family, which we name “*Candidatus* Subterrananammoxibius californiae.” Its genome contains all the necessary apparatus of the core anammox metabolism. We also surveyed the distribution of this family in sediment cores from the Arctic Mid-Ocean Ridge and the Atacama Trench. “*Ca.* Subterrananammoxibiaceae” is confined within the nitrate-ammonium transition zone of the Arctic core where the anammox rate maximum occurs, supporting its anammox metabolic capacity predicted by the genome content. Our results expand the diversity of anammox bacteria and suggest that all resolved families in the *Brocadiales* order harbor anammox bacteria.

## RESULTS AND DISCUSSION

### A novel family in the *Brocadiales* order.

The anammox process has been suggested to account for up to 98% of fixed nitrogen loss in sediments of the Atacama and Kermadec Trenches with water depths of >6,000 m (3). The majority of the anammox bacteria at these sites are closely related to those from shallow oxygen minimum zone waters (3). However, we noticed that there are some minor amplicon sequencing variants (ASVs) in those samples whose phylogenetic affiliations cannot be well resolved based on the phylogenetic analysis of the 16S rRNA gene alone (3).

**FIG 1:**
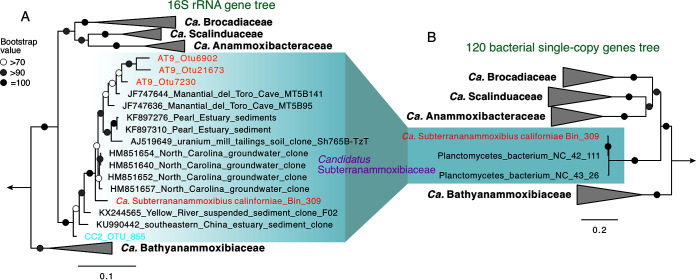
Phylogenetic relationship between “*Candidatus* Subterrananammoxibiaceae” and other known anammox bacteria. (A) Maximum-likelihood phylogenetic trees of bacteria in the *Brocadiales* order based on the 16S rRNA gene. The tree was inferred using IQ-TREE with GTR+F+R5 as the best-fit evolutional model and 1,000 iterations of ultrafast bootstrap analysis. The three OTUs from Atacama Trench sediments are shown in orange, while the OTU from the Arctic core GS13-CC2 is highlighted in blue. (B) Maximum-likelihood phylogenetic trees of bacteria in the *Brocadiales* order based on 120 concatenated bacterial single-copy genes. The tree was inferred using IQ-TREE with LG+F+R8 as the best-fit evolutional model, and 1,000 iterations of ultrafast bootstrap, to assess the robustness of both trees. For both trees, the phylogenetic analyses were done for all major lineages of the *Planctomycetota* phylum, but only branches within the *Brocadiales* order are shown here. The metagenome-assembled genomes (MAGs) recovered in this study from California groundwater samples are highlighted in red. Bootstrap values of >70 are shown with symbols listed in the legend. The scale bars show estimated sequence substitutions per residue.

To identify these unresolved bacterial lineages, we downloaded the 16S rRNA gene amplicon sequencing data of eight sediment cores of the Atacama Trench of up to 8,085 m (28) and reran the operational taxonomic unit (OTU) clustering (400 bp, 97% nucleotide similarity cutoff) and classification. Among the OTUs classified as members of the *Brocadiales* order, we found that three OTUs (OTU_6902, OTU_7230, and OTU_21673) formed a branch separated from the known anammox families *Brocadiaceae*, *Scalinduaceae*, *Bathyanammoxibiaceae*, and *Anammoxibacteraceae* on the phylogenetic tree of the 16S rRNA gene (Fig. 1A). Other sequences falling into this branch were from groundwaters of the Manantial del Toro Cave in Venezuela (29) and North Carolina (30) and sediments of the Pearl Estuary (31), Yellow River (32), and Jiulong River estuary (33). In the above-mentioned literature, these sequences were not classified or named in the original studies, probably due to sequence scarcity. It is worth noting that, currently, there are only three families included in the *Brocadiales* order in the SILVA 138.1 release and that some of the above-mentioned sequences are classified as part of the family 2-02-FULL-50-16-A (renamed “*Ca.* Bathyanammoxibiaceae” in reference 26). However, based on their placements on the 16S rRNA gene phylogenetic tree (Fig. 1A), these sequences represent a separate family parallel to “*Ca.* Bathyanammoxibiaceae” and other known anammox bacterial families.

### A representative metagenome-assembled genome recovered from California groundwater.

Given that this new family is still within the *Brocadiales* order and is sandwiched by known anammox bacterial families (i.e., *Brocadiaceae* and “*Ca.* Bathyanammoxibiaceae”) on the 16S rRNA gene phylogenetic tree, we wished to elucidate whether members of this family have anammox metabolism capacity. Considering their existence in groundwater, estuary sediments, and marine sediments, we focused on these environments in our genome reconstruction effort. We noticed that some novel bacteria affiliated with the *Brocadiales* order exist in the groundwater of a California dairy farm (34). We performed metagenome assembly, binning, and refining (see Materials and Method for details) based on the metagenome sequencing data published in reference 34. We recovered five MAGs affiliated with the *Brocadiales* order: two (Bin_163 and Bin_322) are from the “*Ca.* Brocadiaceae” family, and two (Bin_133 and Bin_313) are from the “*Ca.* Bathyanammoxibiaceae” family, and the last (Bin_309) is suggested by the automatic genome classifier GTDB-Tk (35) to represent a new family based on 120 bacterial single-copy genes. Read mapping and coverage calculation suggest that these five MAGs are mainly present in the three underground samples but not the surface lagoon (see Fig. S1 in the supplemental material), associated with much higher ammonium concentrations (332.8 ppm) than the surface (<0.05 ppm) (34). Given that anammox bacteria in “*Ca.* Brocadiaceae” (10) and “*Ca.* Bathyanammoxibiaceae” (6, 26) have been well established, we focus on the MAG falling into a novel family in the following sections.

Bin_309 is of high quality and is estimated by CheckM to be 94.5% complete with 1.1% redundancy (Table 1). It has 2,837 genes distributed on a total of 72 scaffolds. It is classified as a member of the JACQHT01 family in the latest version of the Genome Taxonomy Database (GTDB 08-RS214), which previously contained only two low-completeness MAGs (*Planctomycetes* NC_groundwater_1106_Ag_S2p5_42_111 [called NC_42_111 here] with 71% completeness and *Planctomycetes* NC_groundwater_466_Ag_B-0.1um_43_26 [NC_43_26] with 68% completeness) recovered from California groundwater (27). Phylogenetic analysis based on the 120 single-copy genes of bacteria supports this classification, in which the three MAGs of JACQHT01 form a separate branch parallel to the other known anammox families (i.e., “*Ca.* Scalinduaceae,” “*Ca.* Anammoxibacteraceae,” “*Ca.* Brocadiaceae,” and “*Ca.* Bathyanammoxibiaceae”) (Fig. 1B). The average amino acid identities (AAIs) between Bin_309 and the two previously available JACQHT01 MAGs (NC_43_26 and NC_42_111) are 98.8% and 99.5%, respectively, indicating that these three MAGs are highly similar and should belong to the same species. In addition, based on the calculated pairwise AAI between all available anammox bacteria genomes, the three JACQHT01 MAGs share only <60% AAI with those from “*Ca.* Bathyanammoxibiaceae” (Fig. S2), suggesting that they represent two different families, consistent with the phylogenetic analysis based on 16S rRNA gene sequences. Moreover, the 16S rRNA gene of Bin_309 shares only 90% nucleotide identity with those of “*Ca.* Bathyanammoxibiaceae” members, lower than the 92% threshold proposed in reference 36, again suggesting that they should represent two separate families.

**TABLE 1:** Summary of anammox bacterial MAGs recovered from the groundwater environment

Measure	“*Ca.* Subterrananammoxibiaceae”			“*Ca.* Bathyanammoxibiaceae”		“*Ca.* Brocadiaceae”	
	Bin_309^*a*^	NC_42_111^*b*^	NC_43_26^*b*^	Bin_313^*a*^	Bin_133^*a*^	Bin_163^*a*^	Bin_322^*a*^
Genome size (Mbp)	3.0	1.6	1.3	1.9	2.4	2.3	3.1
# Scaffolds	72	249	220	264	154	177	596
GC content (%)	42.3	42.6	42.8	50.0	49.5	37.8	41.7
Completeness	95.5%	71.0%	67.6%	87.5%	95.5%	90.1%	70.6%
Redundancy	1.1%	1.1%	1.1%	0.6%	1.1%	1.7%	3.9%
Strain heterogeneity	0.0%	0	100%	66.7%	0.0%	0%	16.7%
N50 of contigs	54,881	7.478	8,820	11,164	22,746	23,220	5,499
# Coding sequences	2,837	1,575	1,164	1,904	2,270	2,128	2,767
Coding density	88.5%	89.1%	89.4%	89.2%	85.7%	84.6%	87.2%
rRNAs	3	1	0	0	0	1	5
tRNAs	47	23	15	26	42	43	22
NCBI accession #							

a MAGs recovered in this study based on the metagenome sequencing data published by Ref. 34.

b MAGs reported in Ref. 27.

Furthermore, Bin_309 has a nearly complete (1,583 base-pair) 16S rRNA gene sequence, which is phylogenetically placed into the novel branch described above (Fig. 1A), indicating that this novel branch corresponds to the family previously delineated JACQHT01. We tentatively rename the f__JACQHT01 family “*Candidatus* Subterrananammoxibiaceae” and the genus g__JACQHT01 “*Candidatus* Subterranammoxibius” to highlight their prevalence in the subsurface environment. We also name Bin_309 “*Candidatus* Subterranammoxibius californiae Bin_309,” to reflect its original discovery. As the only high-quality genome of this taxon so far, “*Ca.* Subterranammoxibius californiae Bin_309” is the type genome for both the “*Ca.* Subterrananammoxibiaceae” family and the “*Ca.* Subterranammoxibius” genus.

### Metabolic functions of “*Ca.* Subterrananammoxibiaceae.”

**FIG 2:**
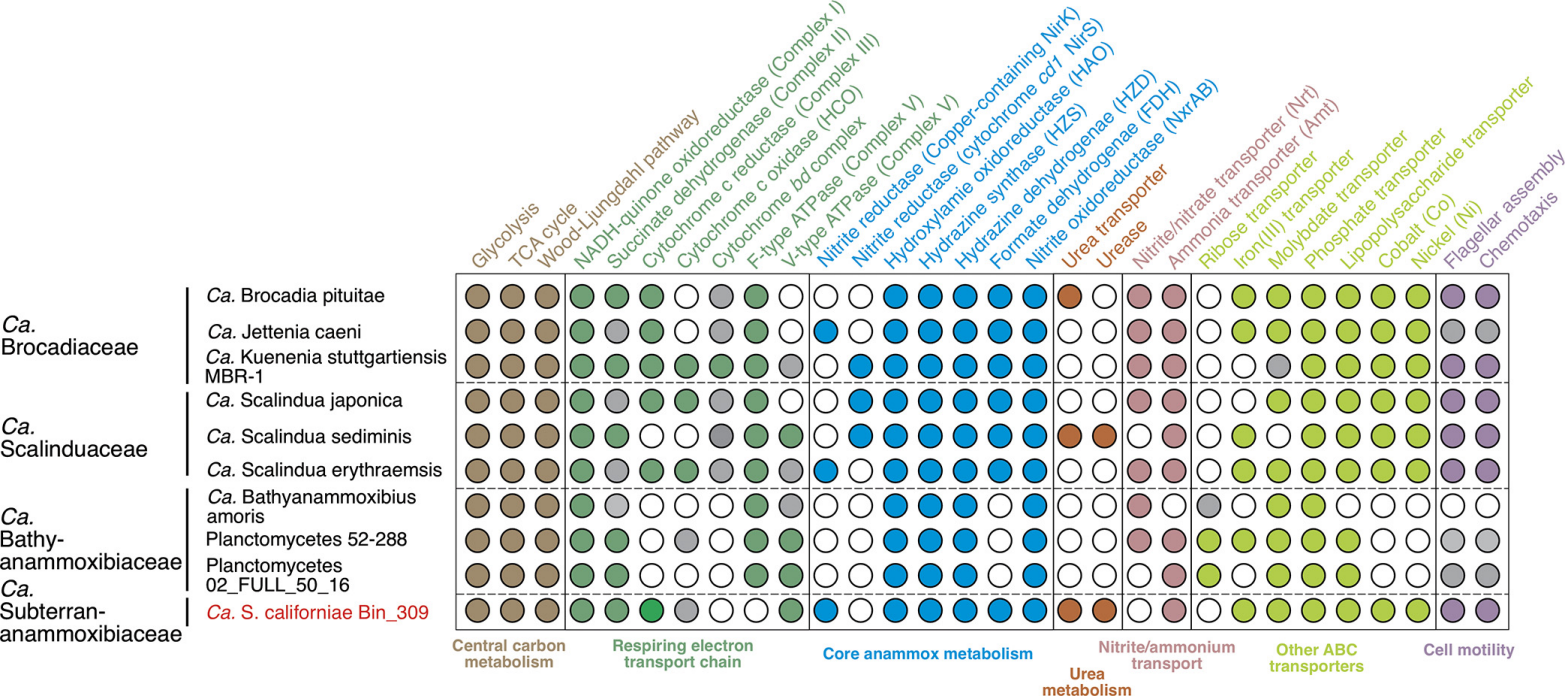
Metabolic potential of anammox bacteria. The family-level affiliations of the representative anammox genomes are shown on the left side. The high-quality MAG of “*Ca.* Subterrananammoxibiaceae” (“*Ca.* S. californiae Bin_309”) recovered in this study is highlighted. Annotations of the other anammox genomes are from reference 26. The filled circles indicate the presence of the full metabolic pathway, open ones indicate the absence, and the gray ones indicate the partial presence of the pathway.

**(i) Hydrazine synthase.** Among the three MAGs of “*Ca.* Subterranammoxibiaceae,” “*Ca.* Subterranammoxibius californiae Bin_309” has the highest completeness level and therefore was selected for the functional and comparative genomic analyses. Hydrazine synthase, one of the most critical enzymes responsible for anammox metabolism, is present in Bin_309 (Fig. 2). The missing genes encoding hydrazine synthase in the other two “*Ca.* Subterranammoxibiaceae” MAGs (i.e., NC_42_111 and NC_43_26) could likely be due to their lower genome completion levels (71% and 68%; Table 1), but we cannot be sure. Although the hydrazine synthase alpha and beta subunits are distributed on the edges of two scaffolds rather than a single operon, phylogenetic trees of both exhibited similar topologies, in which Bin_309 formed a separate branch parallel to other known anammox families (Fig. S3), consistent with the phylogenies inferred from the 16S rRNA gene and 120 single-copy genes (Fig. 1B). This result suggests that the presence of hydrazine synthase genes in “*Ca.* Subterranammoxibius californiae” is not an artifact of the genome reconstruction process. The separation of hydrazine synthase subunits was also previously detected in “*Candidatus* Bathyanammoxibius amoris” (6). In addition to supporting that Bin_309 represents an independent and novel bacterial family, the presence of hydrazine synthase genes in “*Ca.* Subterranammoxibius californiae” also indicates that it has the anammox metabolic capacity.

**(ii) Nitrite metabolism: nitrite reduction.** Nitrite reduction is another critical step of anammox metabolism, in which nitrite reductase can provide NO to the hydrazine synthase (19). Anammox bacteria are thought to use either copper-containing nitrite reductase (NirK) or cytochrome *cd_1_*-containing nitrite reductase (NirS) to carry out this process. However, some known anammox bacteria, especially those from the families “*Ca.* Brocadiaceae” (37) and “*Ca.* Bathyanammoxibiaceae” (26), do not contain either of these two canonical nitrite reductases. This led to the proposal that some anammox bacteria may use one of the hydroxylamine oxidoreductases (HAO) to perform the necessary nitrite reduction process (26, 37, 38). We found a *nirK* gene in Bin_309 and Bin_43_26 (Fig. S4). Phylogenetic analysis suggested that the *nirK* sequences of “*Ca.* Subterrananammoxibiaceae” genomes formed a cluster together with other anammox bacteria (from the families “*Ca.* Scalinduaceae” and “*Ca.* Anammoxibacteraceae”) and nonanammox *Planctomycetota* (Fig. S4). The clusters of anammox bacteria also sandwiched the clusters of nitrite-oxidizing bacteria affiliated with *Nitrospirota* and *Nitrospinota* (Fig. S4), suggesting that *nirK* of these two nitrogen cycling guilds (i.e., anammox bacteria and nitrite-oxidizing bacteria [NOB]) have similar origins or acquisition histories.

**(iii) Nitrite metabolism: nitrite oxidation.** Nitrite oxidoreductase (NXR) is another critical module of the core anammox metabolism, in which NXR can oxidize nitrite to nitrate and provide reducing equivalents (i.e., electrons) to the Wood-Ljungdahl pathway to perform carbon fixation (19). As conserved in all other high-quality anammox bacterial genomes (Fig. 2), Bin_309 and NC_43_26 also encode a complete NXR operon (Fig. 2). Phylogenetic analysis of the NXR alpha subunit (NxrA) showed that the (now five) anammox bacterium families formed five independent clades (Fig. S5). Specifically, NxrA sequences of the two “*Ca.* Subterrananammoxibiaceae” genomes are most closely associated with those of “*Ca.* Scalinduaceae” (Fig. S5). Among the NxrA clades of anammox bacteria, the “*Ca.* Bathyanammoxibiaceae” lineage is placed at the most basal position on the phylogenetic tree, consistent with the proposal that “*Ca.* Bathyanammoxibiaceae” is the basal lineage of anammox bacteria (7, 26). The congruency between the NXR phylogeny and the genome-based phylogeny indicates that the investigated functional traits were present in the anammox bacteria common ancestor and evolved independently in the five anammox bacterium families.

**FIG 3:**
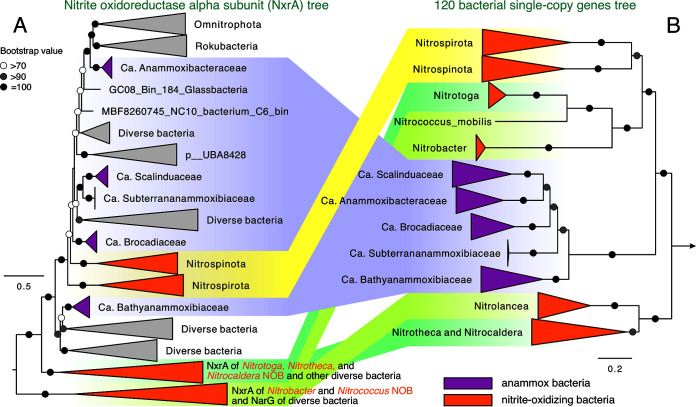
Phylogenetic relationship between lineages of anammox bacteria and nitrite-oxidizing bacteria. (A) Maximum-likelihood phylogenetic tree of nitrite oxidoreductase alpha subunit (NxrA) in anammox bacteria, NOB, and other uncharacterized bacterial genomes. See Fig. S5 for the expanded clades of the five anammox bacterial families. (B) Maximum-likelihood phylogenetic tree of anammox bacteria and NOB based on the 120 bacterial single-copy genes. Bootstrap values of >70 are shown with symbols listed in the legend. The scale bars show estimated sequence substitutions per residue. Lineages of the same phylogenetic affiliation in both trees are highlighted and connected using color bands to better delineate the inconsistencies (e.g., potential horizontal gene transfers) between the two trees.

**(iv) Horizontal transfer of NXR between anammox bacteria and NOB of *Nitrospira* and *Nitrospina*.** Resolving the five anammox bacterial lineages is helpful to delineate the evolutionary history of NXR among lineages of anammox bacteria and nitrite-oxidizing bacteria (NOB). NXR in NOB are not monophyletic, and some horizontal transfer events of the NXR module (39) are thought to be responsible for the spread of this functional trait among lineages in the four bacterial phyla (*Nitrospirota*, *Nitrospinota*, *Proteobacteria*, and *Chloroflexota*) known for oxidizing nitrite aerobically. On the broad phylogenetic tree of NxrA, including all lineages of NOB and anammox bacteria (Fig. 3A), the clades *Nitrospirota* and *Nitrospinota* NOB are sandwiched by the lineages of anammox bacteria, both of which have the periplasmic-facing NXR, while NOB of *Nitrobacter* and *Nitrococcus* (in *Proteobacteria*) and *Nitrolancea* (in *Chloroflexota*) have cytoplasmic-facing NXR. Because NOB of *Nitrospirota* and *Nitrospinota* are from two phyla different from the *Planctomycetota* phylum that harbors anammox bacteria (Fig. 3B), the phylogenetic similarity of NXR between them likely resulted from horizontal gene transfer events, supporting the arguments of references 40 and 41. Based on the available genome information, the origin of anammox bacteria on Earth has been constrained around the Great Oxygenation Event of 2.3 to 2.5 billion years ago (7), while the emergence time of *Nitrospira* and *Nitrospina* NOBs have not yet been well constrained, although an estimate of 830 million years ago (Myr) ago based on a limited number of *Nitrospira* 16S rRNA gene sequences has been suggested for *Nitrospira* NOB (40). An improved understanding of the origins of these NOBs should provide better insights into the spreading routes of NXR across different nitrogen cycling guilds.

**FIG 4:**
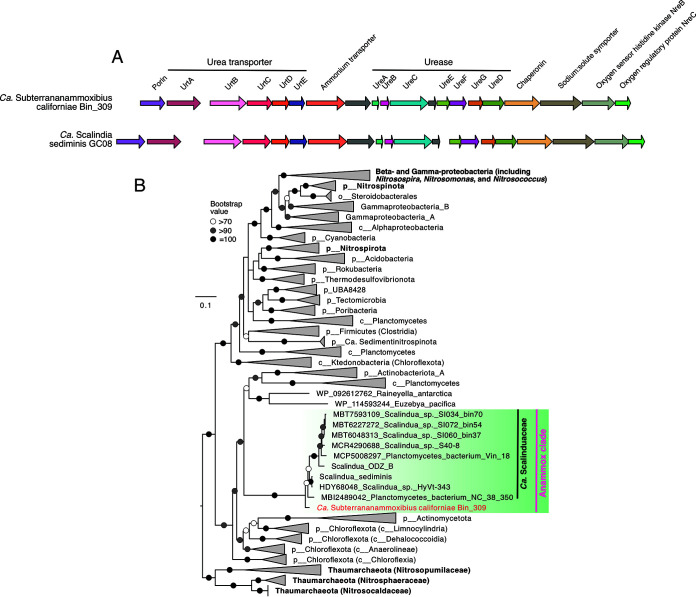
Urease and associated proteins in anammox bacteria. (A) Gene synteny of urease, urea transporter, and other associated genes in “*Ca.* Subterranammoxibius californiae Bin_309” and reference genome “*Ca.* Scalindua sediminis” strain GC08. Genes of the same function are shown in the same color. (B) Maximum-likelihood phylogenetic tree of urease alpha (and catalytic) subunit (UreC). The tree was inferred using IQ-TREE with LG+R8 as the best-fit evolutional model. The 1,000 iterations of ultrafast bootstrap analysis were applied to assess the robustness of both trees. The metagenome-assembled genome (MAG) recovered from California groundwater in this study is shown in red, and the clade formed by anammox bacteria is highlighted by a green box. Functional groups involved in nitrogen cycling are shown in bold. Bootstrap values of >70 are shown with symbols listed in the legend. The scale bar shows the estimated sequence substitutions per residue.

**(v) Urea utilization in anammox bacteria.** “*Ca.* Subterranammoxibius californiae Bin_309” contains the operon of urease and urea-associated proteins (Fig. 4A). The absence of urease in the other two “*Ca.* Subterrananammoxibiaceae” MAGs may be due to the low genomic completions (<71% or lower). The gene arrangements of urease, urea transporter, and associated genes are almost identical to those in “*Candidatus* Scalindua sediminis” (4), an anammox bacterial MAG containing the most complete urease-related genes (Fig. 4A). In both genomes, the urease genes (*ureABCEFGD*) are flanked by the urea ABC transporter genes (*urtABCDE*) and a porin (Fig. 4A). Between them is an ammonium transport gene that is only conserved in the urease-encoding anammox bacteria (Fig. 4A), suggesting that this is a special ammonium transporter of anammox bacteria. This transporter may be involved in transporting the ammonium released from ureolysis into anammoxosome for hydrazine synthesis. Anammox bacteria, except “*Ca.* Bathyanammoxibiaceae,” also contain the ABC transporter of nickel (Fig. 2), the essential cofactor of urease (42), which may help provide sufficient nickel for the maturation of urease in anammox bacteria. The urease genes are also flanked by an operon of the oxygen sensor two-component regulatory system NreB/NreC (Fig. 4A), which regulates the nitrate/oxygen cosensing in facultative bacteria (43, 44). When oxygen is depleted, NreB/NreC in these bacteria activates the expression of the nitrate (*narGHI*) and nitrite (*nir*) reductase operons (43), as well as the putative nitrate transporter gene *narT* (by similarity). The presence of NreB/NreC genes at the proximity of urease genes in anammox bacteria may indicate that their urea transport and lysis may be oxygen sensitive.

Previously, anammox in the wastewater environment (typically dominated by “*Ca.* Brocadiaceae” members) was suggested to be not capable of urea utilization (45). However, analyses of marine anammox have indicated that anammox bacteria of the “*Ca.* Scalinduaceae” family in both marine sediments (4) and oxygen-deficient water columns (21, 46, 47) have the genetic apparatus for urea hydrolysis. This is the first time that anammox bacteria in a terrestrial environment have been revealed to have the potential to degrade urea to ammonium. Phylogenetic analysis of the urease alpha subunit (UreC) showed that anammox bacteria formed a monophyletic cluster separate from other ureolytic bacteria. Anammox UreC sequences have the highest similarities to two actinobacterial isolates (i.e., Raineyella antarctica [48] and Euzebya pacifica [49]) (Fig. 4B). The UreC of Bin_309 fell into a branch distinct from that of the “*Ca.* Scalinduaceae” family, again supporting that Bin_309 represents a separate anammox bacterium family. Consistent with an earlier analysis (4), ureases in anammox bacteria fell into a clade different from that of nitrifiers, such as ammonia-oxidizing archaea and bacteria, and nitrite-oxidizing bacteria (Fig. 4B), suggesting that anammox bacteria should have a different acquisition history than nitrifiers. “*Ca.* Subterrananammoxibiaceae” is the second anammox bacteria family (after “*Ca.* Scalinduaceae”) that is known to harbor members capable of urea hydrolysis.

Urea hydrolysis not only provides extra ammonium to anammox bacteria but may also contribute to the energetic budget. Urea hydrolysis in microbial cells (e.g., Ureaplasma urealyticum) has been demonstrated to be coupled to ATP formation (50, 51) by generating a net increase in intracellular pH, resulting in a proton gradient used to drive proton-dependent ATP synthase. Also, microbes have been observed to grow with urea as the single substrate (50), supporting the concept that urea hydrolysis can provide energy and carbon sources to ureolytic microorganisms.

### “*Ca.* Subterrananammoxibiaceae” in marine sediments.

**FIG 5:**
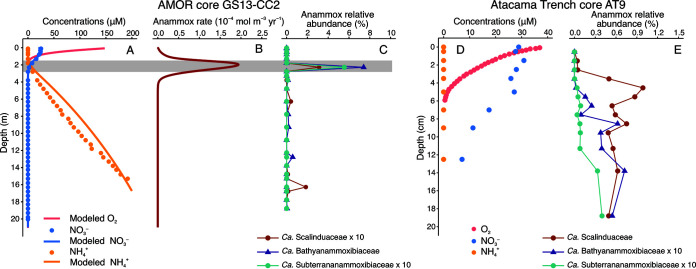
Biogeochemical profiles of two marine sediment cores where “*Ca.* Subterrananammoxibiaceae” are detected. (A to C) Profiles of AMOR core GS13-CC2. (A) Measured (dots) and modeled (lines) depth profiles of oxygen, nitrate, and ammonium. (B) Anammox rate predicted by a reaction-transport model. (C) Relative abundances of three cooccurring putative anammox bacterial families as assessed by 16S rRNA gene amplicon sequencing. The nitrate-ammonium transition zone in this core is highlighted by a gray band. (D and E) Geochemical profiles (D) and distribution of three anammox bacterial families (E) in the Atacama Trench core AT9. The oxygen profile is from reference 55, while the nitrate and ammonium profiles are reported in reference 3. Note that 10-fold values of the relative abundances of two anammox bacterial families in both cores are shown to make them more visible.

Quantitative abundance and environmental context are critical to revealing the redox preference of novel microbial groups such as “*Ca.* Subterrananammoxibiaceae,” which are yet available in the literature. We sought to determine where anammox bacteria of “*Ca.* Subterrananammoxibiaceae” are limited to the terrestrial environment by examining sediment cores previously retrieved from the Arctic Mid-Ocean Ridge (AMOR). In such cores the distribution of relevant nitrogen species can be well resolved (4, 6). We detected the presence of “*Ca.* Subterrananammoxibiaceae” in a 21-m-long core, GS13-CC2, collected from the west flank of the AMOR beneath the Greenland and Norwegian Seas (Fig. S6). “*Ca.* Subterrananammoxibiaceae” in this core is represented by OTU_855, which falls into this family on the phylogenetic tree of the 16S rRNA gene (Fig. 1A). Based on the measured porewater profiles of nitrate and ammonium (Fig. 5A), it features a nitrate-ammonium transition zone (4) around 2.3 m below the seafloor (Fig. 5A). Similar to other AMOR cores described previously in reference 4, this zone harbors the highest anammox reaction rate based on the prediction of a reaction-transport model (Fig. 5B), which can reproduce most of the measured geochemical profiles (Fig. 5A and Fig. S6) when using the model parameters listed in Table S1 and S2. As expected, the relative abundance maxima of anammox the bacterial families “*Ca.* Bathyanammoxibiaceae” (7.4% of the total) and “*Ca.* Scalinduaceae” (0.3%) are detected in the nitrate-ammonium transition zone (Fig. 5C). Importantly, “*Ca.* Subterrananammoxibiaceae” is exclusively present in the nitrate-ammonium transition zone where the anammox reaction occurs (Fig. 5C), although it accounts for only 0.6% of the total community. This distribution pattern provides ecological evidence that “*Ca.* Subterrananammoxibiaceae” should engage in anammox metabolism and is present across both the terrestrial and marine subsurfaces.

“*Ca.* Subterrananammoxibiaceae” members are also present in Atacama Trench core AT9, retrieved from the abyssal seafloor with a water depth of 4,050 m. This family accounts for only <0.04% of the total prokaryotic population (Fig. 5E), while the anammox bacterial communities are dominated by “*Ca.* Scalinduaceaea” (3). Its relative abundance shows a downcore increasing trend toward deeper sediments (Fig. 5E). Whether it shows a preference for the presumed nitrate-ammonium transition zone is still unclear, because the nitrate-ammonium transition zone of this core cannot be reliably resolved based on the available geochemical profiles of nitrate and ammonium (Fig. 5D). Nonetheless, these results suggest that “*Ca.* Subterrananammoxibiaceae” members are exclusive to the Arctic site.

**Conclusion.** We report a new (and the fifth) family in the *Brocadiales* order, “*Ca.* Subterrananammoxibiaceae” and suggest that members of this family are overlooked anammox bacteria. The representative genome, “*Ca.* S. californiae,” contains genes encoding all critical enzymes of anammox metabolism, including nitrite reductase, hydrazine synthase, hydrazine dehydrogenase, and nitrite oxidoreductase. In addition, “*Ca.* S. californiae” also contains urease and therefore may be able to generate extra ammonium and conserve energy from urea hydrolysis. Members of “*Ca.* Subterrananammoxibiaceae” are notably present in various global environments, including groundwater in California and North Carolina, hypersaline cave groundwater in Venezuela, estuary sediments in East and South China, and marine sediments in the Arctic and Pacific Oceans. Their global distribution in both the marine and terrestrial subsurface belies the importance of better understanding their phylogeny and function in future studies. In the Arctic sediment core where geochemical zonation is well resolved, “*Ca.* Subterrananammoxibiaceae,” along with the other two anammox bacterial families, favors the sediment layers where the anammox process prevails, providing ecological evidence of the anammox capacity of this new family. After recognizing this family, it becomes clear that NXR evolved independently with each individual anammox bacterial family, but horizontal gene transfer may have happened between anammox bacteria and nitrite-oxidizing bacteria of *Nitrospira* and *Nitrospina*. Future enrichment and cultivation efforts are needed to provide more physiological and ecological insights into this family.

## MATERIALS AND METHODS

### Mining potential novel anammox bacteria from Atacama Trench sediments.

To discover potential novel anammox bacteria from Atacama Trench sediments, we downloaded the amplicon sequencing data from European Nucleotide Archive using the project number PRJEB33873. The sequencing data were processed using USEARCH (52) following the procedure described previously in reference 4. Briefly, the forward and reverse reads were merged, allowing 1 mismatch and with a minimum length of 400 bp, and the merged reads were used for OTU clustering (and chimera detection and removal) using the cutoff of 97% nucleotide similarity. The OTU sequences were taxonomically classified using CREST (53) with the SILVA 138.1 release (54) as the reference. OTUs classified as members of the *Brocadiales* were extracted and added to the backbone phylogenetic tree of the 16S rRNA gene sequences of major *Planctomycetota* lineages as reported in reference 26, to verify the automatic classification. The phylogenetic placements were also used to group the *Brocadiales* OTUs into putative anammox bacterium families, in which relative abundances were plotted against the sample depth and other geochemical profiles presented in reference 3 and oxygen profiles presented in reference 55.

### Genome binning and refining.

We downloaded the metagenome sequencing data of the four samples (i.e., domestic supply well [DOM], monitoring well 5 [MW5], monitoring well 6 [MW6], and surface lagoon [LAG]) reported in reference 34 from the NCBI database (BioProject accession PRJNA342017). The quality of raw reads (2 × 100-bp paired-end) was first checked using FastQC v0.11.9 (56), and adapter removal and quality-based read trimming were performed using BBDuk as implemented in BBMap (57). The quality-controlled paired-end reads of the four samples were *de novo* coassembled into a single set of contigs using MEGAHIT v1.1.2 (58) with the setting –meta-sensitive (i.e., with k-mers 21, 31, 41, 51, 61, 71, 81, 91, and 99). Contigs larger than 1,000 bp were grouped into genome bins using MaxBin 2 v2.2.5 (59) and MetaBAT v2.15.3 (60) with the default parameters. The resulting MAGs were processed using DAS_Tool v1.1.2 (61) with the default settings, to select MAGs of the best quality for each lineage. The quality of the obtained genome bins was assessed using the option “lineage_wf” of CheckM v1.0.7 (62) and taxonomically classified using GTDB-Tk v2.0.0 (35) with the default settings. MAGs of distant relatedness to known anammox bacteria were subjected to further refinement.

To improve the quality of Bin_309, quality-trimmed reads of MW6 were aligned onto the contigs of the two MAGs using BBMap (57), and the successfully aligned reads were reassembled using SPAdes v3.12.0 (63) with k-mers 21, 33, 55, and 77. After the removal of contigs shorter than 1,000 bp, the resulting scaffolds were visualized and manually rebinned using gbtools v2.6.0 (64), based on the GC content, taxonomic assignments, and differential coverages of contigs across multiple samples. To generate the input data of the genome refinement, coverages of contigs in each sample were determined by mapping trimmed reads onto the contigs using BBMap v37.61 (57). Taxonomy classifications of contigs were assigned using BLASTn (65) according to the taxonomy of the single-copy marker genes in contigs. Small-subunit (SSU) rRNA sequences in contigs were identified using Barrnap (66) and classified using VSEARCH (56) with the SILVA 132 release (57) as the reference. The quality of the refined Bin_309 and Bin_313 was checked using the CheckM v1.0.7 “lineage_wf” command again, based on the *Planctomycetes* marker gene set.

### Genome annotation.

Bin_309 was annotated with the two MAGs included in the family JACQHT01 in the GTDB release 08-RS214 (https://gtdb.ecogenomic.org/). Genes in these genomes were predicted using Prodigal (67). Genome annotation was conducted using Prokka v1.13 (68), eggNOG (69), and BlastKoala (70) using the KEGG database. The functional assignments of genes of interest were also confirmed using BLASTp (66) against the NCBI RefSeq database. The metabolic pathways were reconstructed using KEGG Mapper (71).

### Phylogenetic analyses.

To pinpoint the phylogenetic placement of Bin_309 and the relative genomes in the family JACQHT01, we performed phylogenetic analyses for them together with high-quality genomes of the *Planctomycetes* phylum that was included in the GTDB release 08-RS214. The 120 single-copy genes were identified, aligned, and concatenated using GTDB-Tk v2.0.0 (35) with the “classify_wf” command. The maximum-likelihood phylogenetic tree was inferred based on this alignment using IQ-TREE v1.5.5 (72) with LG+F+R7 as the best-fit model selected by ModelFinder (73) and 1,000 ultrafast bootstrap iterations using UFBoot2 (74). To provide support to this phylogenomic tree, we also performed the phylogenomic analysis based on the 14 syntenic ribosomal proteins (rpL-2, -3, -4, -5, -6, -14, -16, -18, and -22 and rpS-3, -8, -10, -17, and -19) that have been demonstrated to undergo limited lateral gene transfer (75). These selected proteins were identified in Anvi’o v7.1 (67) using hidden Markov model (HMM) profiles and aligned individually using MUSCLE (76). Alignment gaps were removed using trimAl (77) with the “automated” mode. Individual alignments of ribosomal proteins were concatenated. The maximal likelihood phylogenetic tree was reconstructed using IQ-TREE v1.5.5 (72) with LG+R7 as the best-fit model.

A maximum-likelihood phylogenetic tree based on 16S rRNA genes was also constructed to highlight the phylogenetic placement of the “*Ca.* Subterrananammoxibiaceae” family in the *Planctomycetes* phylum. To expand this family on the tree beyond the available genomes, the three OTUs from the amplicon sequencing of the hadal Trench sediments (3) and their close relatives identified via BLASTn (78) in the NCBI database were also included. Sequences were aligned using MAFFT-LINSi (79), and the maximum-likelihood phylogenetic tree was inferred using IQ-TREE v1.5.5 (72) with GTR+F+R3 as the best-fit substitution model and 1,000 ultrafast bootstraps, following the procedure described above.

For the phylogenies of hydrazine synthase alpha subunit (HzsA) and hydrazine synthase beta subunit (HzsB), the sequences of the newly recovered MAGs were added to the backbone sequences compiled in reference 26. All sequences were aligned using MAFFT-LINSi (79) and trimmed using trimAl (77) with the “automated” mode. The maximum likelihood phylogenetic trees were inferred using IQ-TREE v1.5.5 following the procedure described above.

For the phylogeny of NxrA encoding the nitrite oxidoreductase alpha subunit, the sequence of Bin_309 was used as the query in the BLASTp (78) search in the NCBI database (>50% similarity and E-value of 10^−6^) to identify its close relatives. These sequences were aligned using MAFF-LINSi (79) with sequences compiled in reference 26. The alignment was then trimmed using trimAl (77) with the “automated” mode. Maximum likelihood phylogenetic trees were reconstructed using IQ-TREE v1.5.5 (72) with the LG+C20+F+G substitution model and 1,000 ultrafast bootstraps.

For the phylogeny of copper-containing nitrite reductase (NirK) and urease alpha subunit (UreC), reference sequences were mainly extracted from references 80 and 4, respectively. Additional sequences were obtained from NCBI using BLASTp with the anammox NirK and UreC sequences as the queries. After alignment using MAFF-LINSi (79) and trimming using trimAl (77), the phylogenetic tree was inferred using IQ-TREE v1.5.5 (72) with the LG+C20+F+G substitution model and 1,000 ultrafast bootstraps. The two clades (clade I and clade II) of the NirK phylogenetic tree were defined following H. Decleyre et al. (80).

### Data availability.

All sequencing data used in this study are available in the NCBI Sequence Read Archive under the project number PRJNA947605. The 16S rRNA gene amplicon sequencing data of core GS13-CC2 are deposited in the NCBI Short Reads Archive under the project number PRJNA991510.
