# Supplementary material for: “Candidatus Subterrananammoxibiaceae,” a New Anammox Bacterial Family in Globally Distributed Marine and Terrestrial Subsurfaces
Source: Appl Environ Microbiol. 2023 Jul 20;89(8):e00800-23. doi: 10.1128/aem.00800-23 (PMC10467342; doi:10.1128/aem.00800-23)
Supplement: Supplemental file 1 — Fig. S1 to S6 and Tables S1 to S2. Download aem.00800-23-s0001.docx, DOCX file, 2.8 MB [file aem.00800-23-s0001.docx]

**Supplementary Figures**


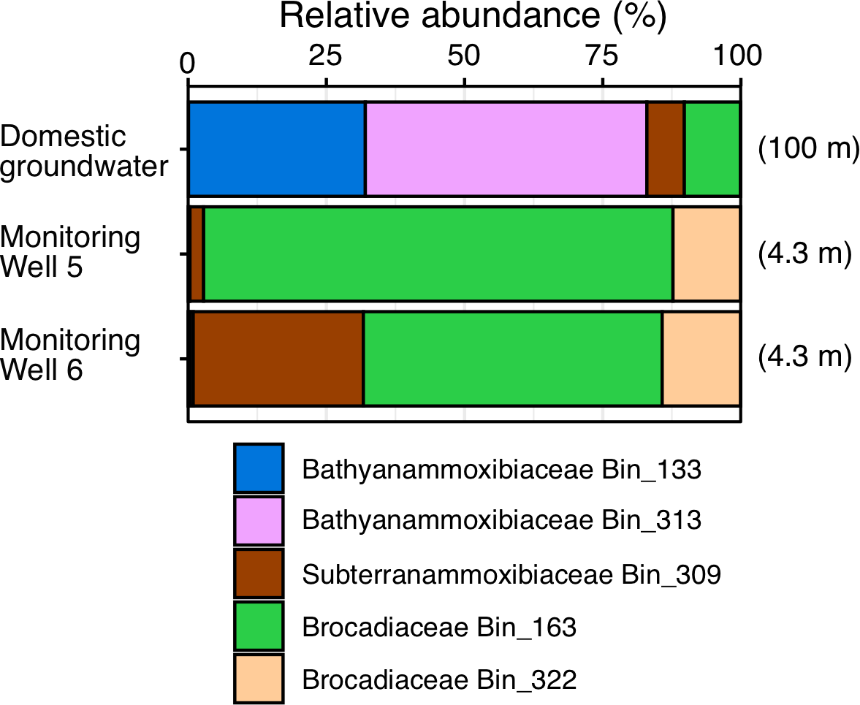


**Figure S1. Genome-resolved anammox bacterial community composition in three groundwater types.**


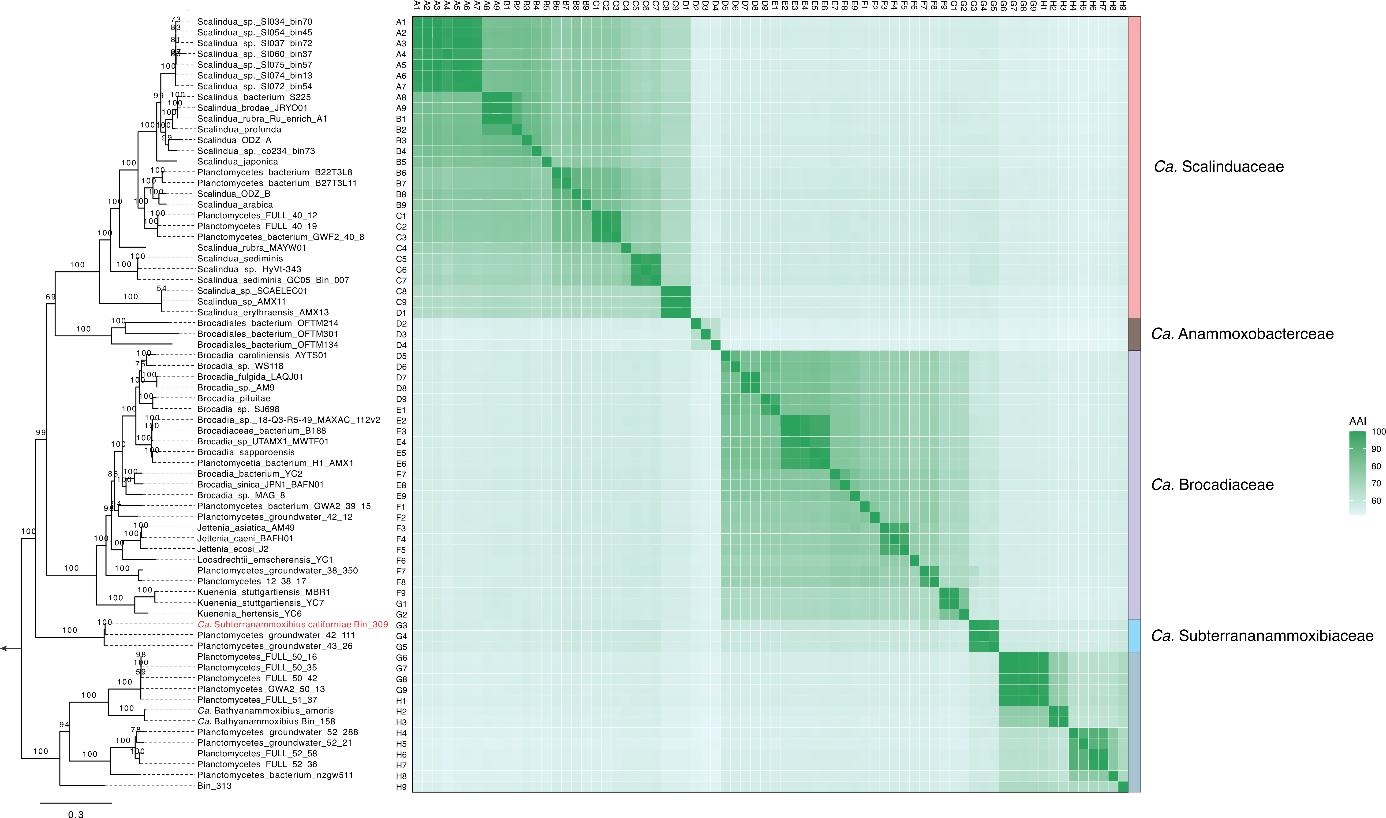


**Fig. S2. Average amino acid identity (AAI) between different families of putative anammox bacteria genomes.**

**
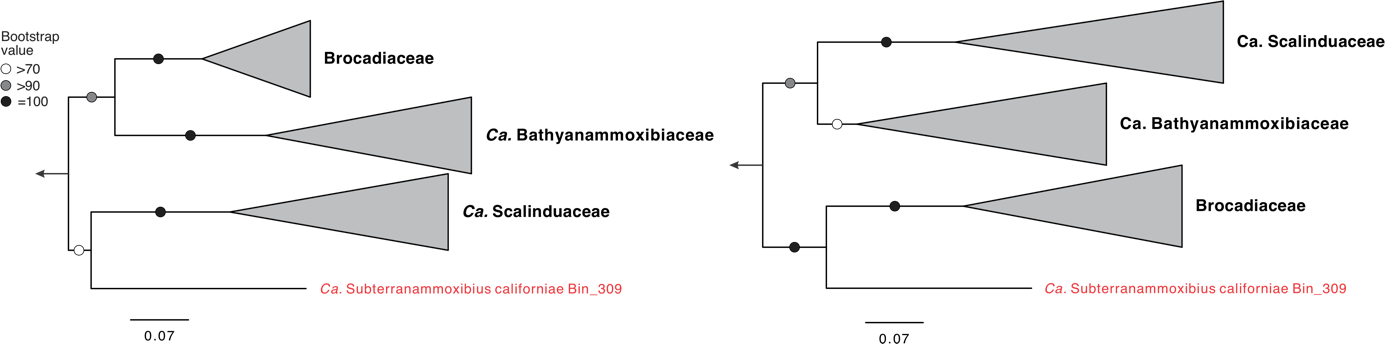
**

**Fig. S3. Maximum-likelihood phylogenetic trees of hydrazine synthase alpha (A) and beta (B) subunits between *Candidatus* Subterranammoxibiaceae and other known anammox bacteria.** Both trees were inferred using IQ-TREE with LG+I+G4 as the best-fit evolutional model. 1,000 times of ultrafast bootstrap iteration was applied to assess the robustness of both trees. Both trees are rooted to sequences of anammox bacteria in the *Ca.* Anammoxibacteraceae family. The metagenome-assembled genome (MAG) recovered from California groundwater in this study is highlighted in green. Bootstrap values of >70 are shown with symbols listed in the legend. The scale bars show estimated sequence substitutions per residue.


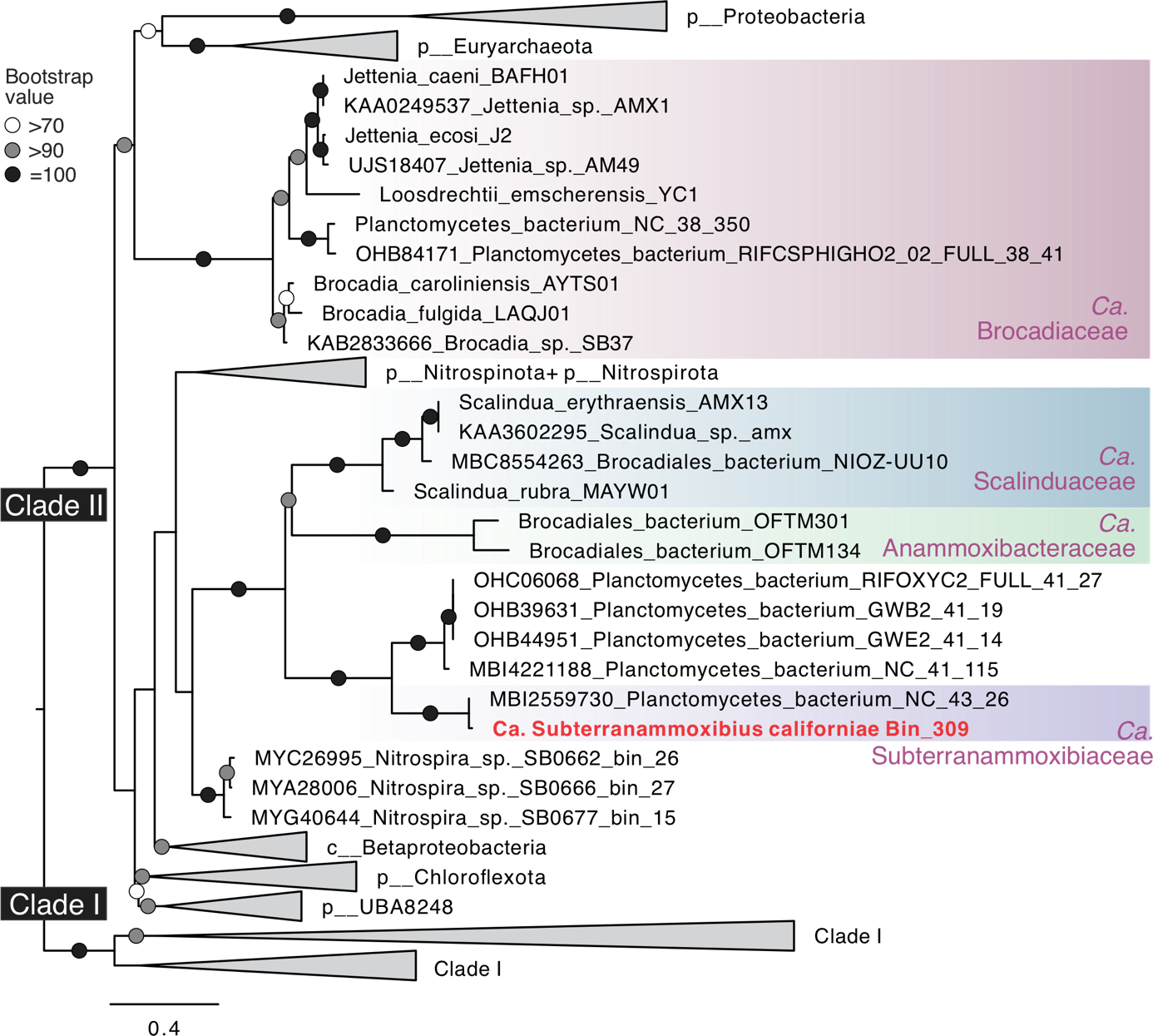


**Figure S4. Maximum-likelihood phylogenetic tree of copper-containing nitrite reductase (NirK).** The tree is inferred using IQ-TREE with LG+R8 as the best-fit evolutional model. 1,000 times of ultrafast bootstrap iteration was applied to assess the robustness of the trees. The clades formed by four families of anammox bacterial are highlighted by various colors of boxes, whereas so far members of the other anammox family, *Ca.* Bathyanammoxibiaceae, are not known to contain NirK. Clades formed by prokaryotes other than anammox bacteria are collapsed for readability. Bootstrap values of >70 are shown with symbols listed in the legend. The scale bar shows estimated sequence substitutions per residue.


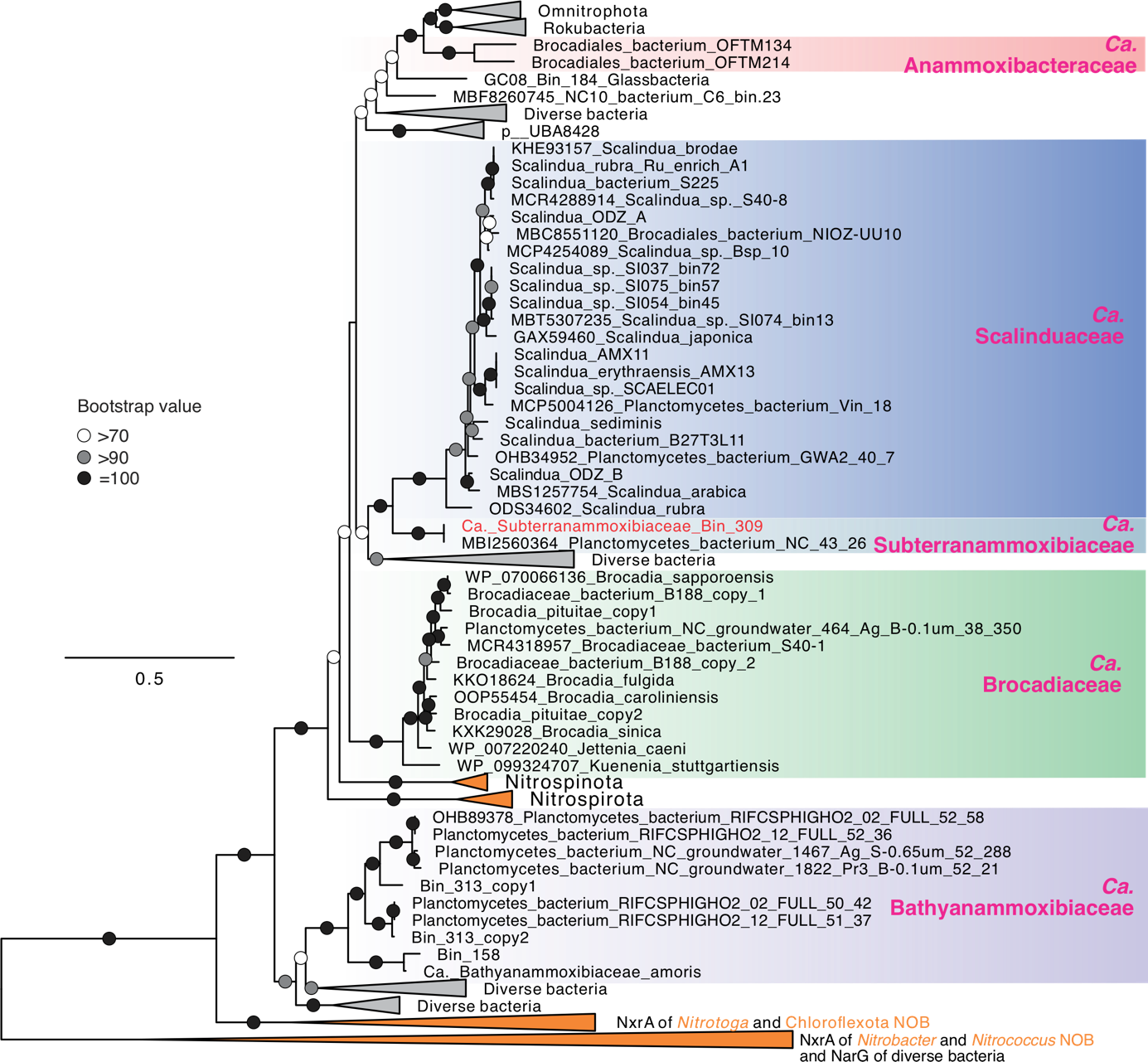


**Figure S5. Maximum-likelihood phylogenetic tree of nitrite oxidoreductase alpha subunit (NxrA).** The tree was inferred using IQ-TREE with LG+R8 as the best-fit evolutional model. 1,000 iterations of ultrafast bootstrap was applied to assess the robustness of the trees. The metagenome-assembled genome (MAG) recovered from California groundwater in this study is shown in red, and the clades formed by anammox bacteria are highlighted by various colors of boxes. The clades of known nitrite-oxidizing bacteria are shown in orange. Bootstrap values of >70 are shown with symbols listed in the legend. The scale bar shows estimated sequence substitutions per residue.


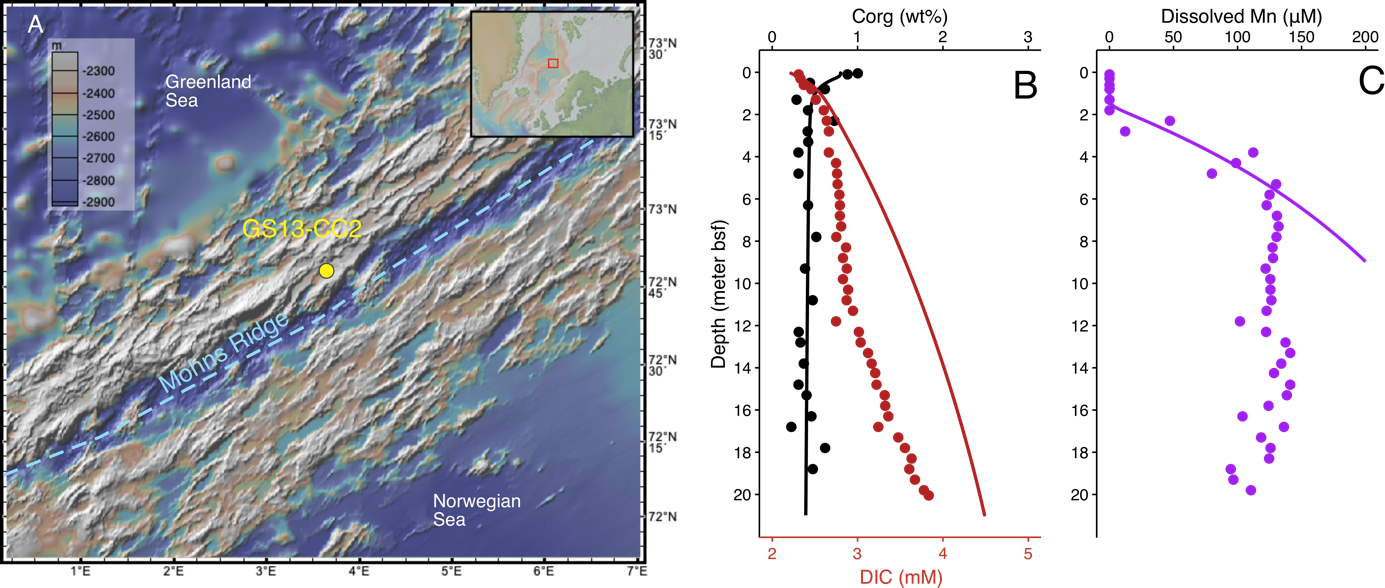


**Fig. S6. Bathymetric map showing the sampling location of core GS13-CC2.** The insert shows the sampling location in the Arctic Mid-Ocean Ridge area. This map was generated with GeoMapApp v. 3.6.14 ([www.geomapapp.org](http://www.geomapapp.org)), using the default Global Multi-Resolution Topography Synthesis basemap. (B-C) Depth profiles of total organic carbon (Corg), dissolved inorganic carbon (DIC) (B), and dissolved Mn (C) in core GS13-CC2. The dots indicate measured values, while the lines represent reaction-transport model simulations.

Table S1. Species and boundary conditions (BC) at the sediment-water interface (SWI) used in the reaction-transport model

| **Name** | **Symbol** | **BC SWI Type (Unit)** | **GS13-CC2** |
| --- | --- | --- | --- |
| Total organic carbon flux | CH_2_O | Flux (mol m^-2^ yr^-1^) | 8E-3 |
| Oxygen | O_2_ | Concentration (µM) | 150 |
| Ammonium | NH_4_^+^ | Concentration (µM) | 0.1 |
| Nitrate | NO_3_^–^ | Concentration (µM) | 21 |
| Manganese | Mn(II) | Concentration (µM) | 0.1 |
| DIC | HCO_3_^–^ | Concentration (mM) | 2.2 |

**Table S2. Parameter values used in the reaction-transport model**

| **Name** | **Symbol** | **Unit** | **GS13-CC2** |
| --- | --- | --- | --- |
| Sediment domain | L | cm | 4000 |
| Solid burial velocity at compaction | ω | cm ky-1 | 2.0 |
| TOC degradation constant C_1_ | kfox | 1 yr^-1^ | 3.4E-5 |
| TOC degradation constant C_2_ | kfox2 | 1 yr^-1^ | 1E-6 |
| Nitrification rate constant | *k*_4_ | mM^-1^ yr^-1^ | 150 |
| Mn oxidation rate constant | *k*_5_ | mM^-1^ yr^-1^ | 110 |
| Anammox rate constant | *k*_6_ | mM^-1^ yr^-^ | 10 |
| Bioturbation coefficient | *D*_b,0_ | cm yr^-1^ | 1 |
| Biomixing half depth | z_mix_ | cm | 3 |
| Biomixing attenuation | Z_att_ | cm | 3 |
| Bioirrigation coeffcient | *α*_0_ | yr^-1^ | 0 |
| *R*_1_ O_2_ inhibition concentration | *h*_1_ | µM | 10 |
| *R*_2_ NO_3_^-^ inhibition concentration | *h*_2_ | µM | 20 |
| *R*_3_ MnO_2_ inhibition concentration | *h*_3_ | µmol g^-1^ | 5 |
| Porosity at sediment surface | φ_0_ | -- | 0.8 |
| Porosity at infinite depth | φ_∞_ | -- | 0.6 |
| Porosity attenuation coefficient | *α*_0_ | cm^-1^ | 0.01 |
